# Next-Generation SINE Compound KPT−8602 Ameliorates Dystrophic Pathology in Zebrafish and Mouse Models of DMD

**DOI:** 10.3390/biomedicines10102400

**Published:** 2022-09-26

**Authors:** Katherine G. English, Andrea L. Reid, Adrienne Samani, Gerald J. F. Coulis, S. Armando Villalta, Christopher J. Walker, Sharon Tamir, Matthew S. Alexander

**Affiliations:** 1 Department of Pediatrics, Division of Neurology at Children’s of Alabama the University of Alabama at Birmingham, Birmingham, AL 35233, USA; 2 Department of Physiology and Biophysics, University of California-Irvine, Irvine, CA 92697, USA; 3 Institute for Immunology, University of California-Irvine, Irvine, CA 92967, USA; 4 Karyopharm Therapeutics, Newton, MA 02459, USA; 5 UAB Center for Exercise Medicine (UCEM), Birmingham, AL 35205, USA; 6 Department of Genetics, University of Alabama at Birmingham, Birmingham, AL 35233, USA; 7 UAB Civitan International Research Center (CIRC), Birmingham, AL 35233, USA; 8 UAB Center for Neurodegeneration and Experimental Therapeutics (CNET), Birmingham, AL 35294, USA

**Keywords:** DMD, SINE compound, KPT−8602, inflammation

## Abstract

Duchenne muscular dystrophy (DMD) is a progressive, X-linked childhood neuromuscular disorder that results from loss-of-function mutations in the DYSTROPHIN gene. DMD patients exhibit muscle necrosis, cardiomyopathy, respiratory failure, and loss of ambulation. One of the major driving forces of DMD disease pathology is chronic inflammation. The current DMD standard of care is corticosteroids; however, there are serious side effects with long-term use, thus identifying novel anti-inflammatory and anti-fibrotic treatments for DMD is of high priority. We investigated the next-generation SINE compound, KPT−8602 (eltanexor) as an oral therapeutic to alleviate dystrophic symptoms. We performed pre-clinical evaluation of the effects of KPT−8602 in DMD zebrafish (*sapje*) and mouse (D2-*mdx*) models. KPT−8602 improved dystrophic skeletal muscle pathologies, muscle architecture and integrity, and overall outcomes in both animal models. KPT−8602 treatment ameliorated DMD pathology in D2-*mdx* mice, with increased locomotor behavior and improved muscle histology. KPT−8602 altered the immunological profile of the dystrophic mice, and reduced circulating osteopontin serum levels. These findings demonstrate KPT−8602 as an effective therapeutic in DMD through by promotion of an anti-inflammatory environment and overall improvement of DMD pathological outcomes.

## 1. Introduction

Duchenne muscular dystrophy (DMD) is a progressive X-linked neuromuscular disorder that affects 1:5000 live male births worldwide, making it the most common childhood neuromuscular disorder [1]. DMD results from mutations in the *DYSTROPHIN* gene, which prevent the production of functional dystrophin protein [2]. DMD patients develop progressive muscle weakness, respiratory and cardiac issues, and loss of ambulation typically by their teenage years. Much of DMD pathology is attributed to the increased permeability of the sarcolemma due to the loss of membrane stability without dystrophin. Consequentially, this leads to muscle cell death, which triggers immune cell infiltration into the muscle and chronic inflammation, a major driver of DMD disease progression and disease pathologies [3,4,5,6]. Although corticosteroids are the current standard of care, there are many studies that report the long-term use of these drugs can cause unwanted side effects, such as excessive weight gain, hypertension, mood alteration, and increased risk of bone fractures [7,8,9,10]. Thus, investigating and identifying new anti-inflammatory DMD therapeutic options with less severe side effects are critical.

A key element to improving DMD pathology is to control the inflammatory response, which is not currently addressed by gene therapies. Prior to muscle weakness, membrane instability of DMD myofibers leads to rapid cycles of degeneration and regeneration [11]. With asynchronous regeneration, myofibers enter the necrotic stage at different times promoting a constant pro-inflammatory micro-environment, marked by the release of cytokines such as CCL22 and CCL14. In the DMD pro-inflammatory state, damaged myofibers release high mobility group box (HMGB1) which binds Toll-like Receptor 4 (TLR4) to activate innate immunity and chronic inflammation [12,13]. A hallmark feature of chronically inflamed dystrophic muscle is the accumulation of M1 and M2 macrophages that contribute to dystrophic pathology by promoting myofiber damage and fibrosis, respectively [14].

Inflammation is regulated in part by the trafficking of RNAs encoding inflammatory mediators that are elevated in DMD to the cytoplasm through the nuclear pore complex (NPC) [15]. One crucial component of the NPC is the nuclear export protein exportin 1 (XPO1). Although XPO1 inhibitors were developed as a treatment strategy for many types of cancer including leukemia and lymphoma, they also possess anti-inflammatory properties [16,17,18,19,20,21,22]. The first XPO1 inhibitor discovered was leptomycin B, which was extracted from a strain of *Streptomyces* and blocks nuclear export [23,24]. Selective Inhibitor of Nuclear Export (SINE) compounds bind reversibly to XPO1 allowing for therapeutic benefit with fewer side effects and an increased dosing regimen, as compared to leptomycin B, which forms an irreversible bond [16,25,26]. A synthetically designed group of inhibitors referred to as selective inhibitor of nuclear export (SINE) compounds have been developed, including selinexor/XPOVIO (KPT−330), verdinexor (KPT−335), KPT−350, and KPT−8602 (eltanexor) to directly block XPO1 nuclear export function. KPT−350, now renamed BIIB100, is currently in clinical development for Amyotrophic Lateral Sclerosis (ALS) patients (A Study to Evaluate the Safety, Tolerability, Pharmacokinetics, and Pharmacodynamics of BIIB100 Administered Orally to Adults with Amyotrophic Lateral Sclerosis; ClinicalTrials# NCT03945279). KPT−8602 is currently in a clinical trial for relapsed and refractory cancers (Study of the Safety, Tolerability and Efficacy of KPT−8602 in Participants With Relapsed/Refractory Cancer Indications; ClinicalTrials# NCT02649790).

Our lab previously demonstrated the beneficial effects of KPT−350 treatment in DMD zebrafish and mice. KPT−350 reduced the severity and delayed onset of dystrophic pathologies in both animal models [27]. Pre-clinical animal models treated with KPT−8602 have demonstrated reduced penetration of the blood–brain barrier and increased tolerability allowing for daily dosing. The efficacy of KPT−8602 treatment has been validated in different cancer types, similar to KPT−350 and has proven tolerable in human patients in a Phase 1/2 clinical trial for colorectal cancer. This clinic-ready oral compound is uniquely suited to a pediatric indication such as DMD [28,29,30,31,32]. Here, we sought to evaluate KPT−8602 in our DMD zebrafish and mouse models to determine if KPT−8602 had therapeutic efficacy in blocking or ameliorating dystrophic pathologies.

## 2. Materials and Methods

### 2.1. Zebrafish Care and KPT−8602 Dosing Experiments

Wild type (*AB* strain) and *sapje* (*dmdta222a* backcrossed to the *AB* background over 10 generations), were maintained at University of Alabama at Birmingham (UAB) aquatics facility under standard housing and feeding conditions (IACUC protocol number 20320). Zebrafish were assessed using birefringence for effects of KPT−8602 treatment as previously described [33,34]. Briefly, *sapje* heterozygotes were mated and the resulting embryos were incubated in the treatment compounds starting at 1 day-post fertilization (dpf). According to Mendelian genetics, approximately 25% of embryos should be homozygous for the *sapje* mutation and display a phenotype though some deviation from this ratio is predicted. Treatments assessed in this assay were vehicle (KPT−8602 formulating agent), KPT−8602 (2.5 µM), and aminophylline (2.5 µM; positive control). Drug compounds were changed every other day until 5 dpf. Larvae (5 dpf) were assessed for birefringence and were categorized as either unaffected or affected (represented as a percentage of the clutch). This was repeated with three (*n* = 3) independent experimental clutches, with at least twenty larvae (*n* = 20) for each trial per treatment group.

### 2.2. Mice

WT (*DBA/2J* strain; stock number 000671) and D2-*mdx* (DMD model of *DBA/2J*; stock number 013141) one month old male mice were originally purchased from Jackson Labs (Bar Harbor, ME, USA) and maintained at UAB under standard housing and feeding conditions (IACUC protocol 21485). All mice were housed under sterile, pathogen-free conditions with ad libitum access to food and water.

### 2.3. KPT−8602 Drug Treatment in Mice

KPT−8602 was delivered orally by mixing the drug compound in peanut butter (Jif Creamy; J.M. Smucker Company; Orrville, OH, USA) pellets as previously described [35]. KPT−8602 drug was obtained directly from Karyopharm Therapeutics and synthesized by Piramal Pharma Solutions (Lexington, KY, USA). The peanut butter pellets contained either vehicle or KPT−8602 (5 mg/kg body weight) and was molded in pellets using 1 mm^3^ squares in plastic mold (CAT#106A; Ted Pella, Redding, CA, USA). Pellets were frozen at −80 °C for at least 4 h to allow for solidification. Mice began treatment regimen at 8 weeks of age and were treated for 8 weeks. Mice were either given vehicle pellets or KPT−8602 (5 mg/kg) pellets for 3 times per week, or given KPT−8602 (5 mg/kg) pellets 5 times per week. Each mouse was given 15 min to consume the peanut butter pellet. After 8 weeks of treatment, mice were assessed in an open field test, followed by euthanization and tissue harvest.

### 2.4. Open Field Test–Basal Activity Tracking

Mice were placed individually in box arenas (60 cm × 60 cm) to monitor basal activity, in terms of total distance traveled and average velocity. Mice were monitored for 5 min in the arena and movements were tracked and analyzed by EthoVision XT Version 15 (Noldus, Leesburg, VA, USA).

### 2.5. Histological Analysis

Tibialis anterior (TA) muscles were immersed in 10% neutral-buffered formalin (CAT#: HT501128, Sigma-Aldrich, St. Louis, MO, USA) for 24 h and subsequently embedded and sectioned in paraffin blocks. Immunostaining was performed on sectioned muscle tissue using flow cytometry validated antibodies against CD206 (Abcam, Waltham MA, USA; ab64693), Ki67 (Abcam; ab15580), and F4–80 (ThermoFisher, Waltham, MA, USA; 14–4801−82). Hematoxylin and Eosin stained histology was assessed for centralized myonuclei (manual counts of at least 300 fibers per muscle section, 5 muscle sections per animal). Fiber size was analyzed using ImageJ software to measure around each fiber to produce a fiber size frequency distribution curve for each animal [36].

### 2.6. Muscle Single-Cell Suspension

Immune cells from a single quadriceps were isolated as previously described with some modifications [37]. Briefly, the quadriceps from KPT−8602 treated mice and control mice were minced and then digested in 0.2 mg/mL collagenase *P* solution supplemented with 20 μg/mL of DNase for two rounds of 20 min enzymatic digestion. The muscle suspension is then sequentially filtered through a 40 and 70 µm filter-basket. Following a final suspension using a 35-µm strainer mesh, immune cells are counted and stained for flow cytometry analysis.

### 2.7. Flow Cytometry Analysis

Flow cytometry analysis of muscle single-cell suspension were performed as described previously [37]. Briefly, to discriminate between live and dead cells, cells are resuspended in 100 μL of Zombie NIR viability dye (1:1000 in 1X PBS; 423105; BioLegend, San Diego, CA, USA) for 15 min on ice while protected from light. Fc receptor blocking of muscle single-cell suspensions was performed by incubating cells with an anti-CD16/32 antibody (clone 2.4G2) prior to staining. Single-cell suspensions were stained with a panel of antibodies against several cell surface antigens to identify macrophages (CD11b and F4/80), or eosinophils (Siglec F). Analysis was performed on live cells on a BD FACSAria Fusion flow cytometer with FACSDiva software (BD Biosciences). Post-acquisition analysis was performed using the FlowJo software version 10.8 (BD Biosciences).

### 2.8. Osteopontin ELISA

A commercial osteopontin (OPN) ELISA kit was purchased (CAT# MOST00, R&D Systems; Minneapolis, MN, USA) and the corresponding protocol was followed. Whole blood serum samples obtained from mouse tail vein bleeds prior to euthanization were added to wells that were pre-coated with OPN polyclonal antibodies. After 1 h of incubation and four washes, OPN conjugate was added to each well. After an additional 2 h of incubation and four additional washes, substrate solution was added. Stop solution was added 30 min later, and the plate was immediately read at 450 nm and 540 nm. Using the standards provided with the assay, OPN concentration (pg/mL) was extrapolated for each sample.

### 2.9. Statistical Analysis

All statistical analysis was performed using GraphPad Prism 9 (GraphPad Software; San Diego, CA, USA). Statistical tests used are described in each figure legend. Unless otherwise stated, statistical analysis between two cohorts used a two-tailed T-test, normality was assessed utilizing a histogram and Q-Q plots for normal distribution, and Levene’s test for homogeneity of variance. Analysis across three or more groups used a one-way ANOVA with a Tukey’s HSD (honest significant difference) test, the residuals were assessed with histograms, Q-Q plots, boxplots, and a Shapiro–Wilks test for normality. An a priori hypothesis stated that *p* < 0.05 is considered significant, with * *p* < 0.05, ** *p* < 0.01, *** *p* < 0.001, and **** *p* < 0.0001.

## 3. Results

### 3.1. KPT−8602 Treatment Reduced Dystrophic Muscle Pathology in Zebrafish Model of DMD

**Figure 1:**
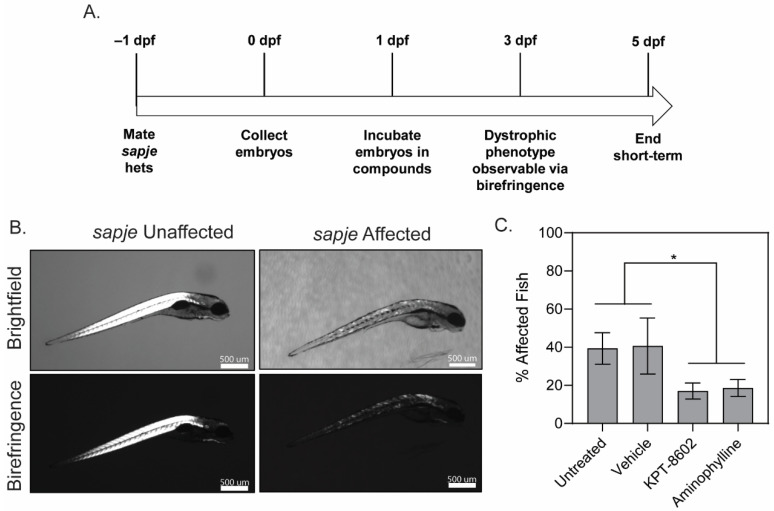
KPT−8602 improves dystrophic muscle pathology in zebrafish larvae. (**A**) Zebrafish drug dosing timeline; (**B**) Representative images of brightfield and birefringence for both unaffected (WT) and affected (*sapje*) zebrafish larvae. Scale bars represent 500 μm; (**C**) Graph summarizing the birefringence score (in percentage of affected/unaffected over total number of larvae assessed in the clutch) across untreated, vehicle-treated, KPT−8602-treated and aminophylline-treated (positive control) fish. KPT−8602 and aminophylline treatment significantly reduced the number of affected fish compared to untreated and vehicle-treated fish (data is represented as mean ± SEM, *n* = 60 per cohort, across 3 independent trials of *n* = 20 each; * *p* < 0.05; one-way ANOVA with Tukey’s HSD test).

The *sapje* DMD model of zebrafish larvae were treated with either 2.5 µM KPT−8602, 2.5 µM aminophylline (positive control), or vehicle control starting at 1 dpf (Figure 1A). Dorsal muscle architecture and integrity of fish larvae were assessed using polarized light in the birefringence assay. When the rigid architecture of skeletal muscle is disrupted, the polarized light is not refracted giving the muscle a patchy or dark appearance due to light passing through the disorganized myofibers [33]. The zebrafish dorsal muscle is significantly affected in *sapje* mutants, which can be clearly observed by the overall disruption of dorsal myofibers shown in the birefringence assay (Figure 1B). Representative bright field and birefringence images from each drug treatment including correction by KPT−8602 and aminophylline are shown (Supplemental Figure S1). KPT−8602 treatment significantly reduced the number of affected larvae, similar to the positive control compound, aminophylline (Figure 1C). KPT−8602 treatment had no effect on basal activity in *sapje* zebrafish larvae at 6 dpf (Supplemental Figure S2).

### 3.2. D2-Mdx Mice Treated with 5x/Week KPT−8602 Had Significantly Improved Activity and Myofiber Size

**Figure 2:**
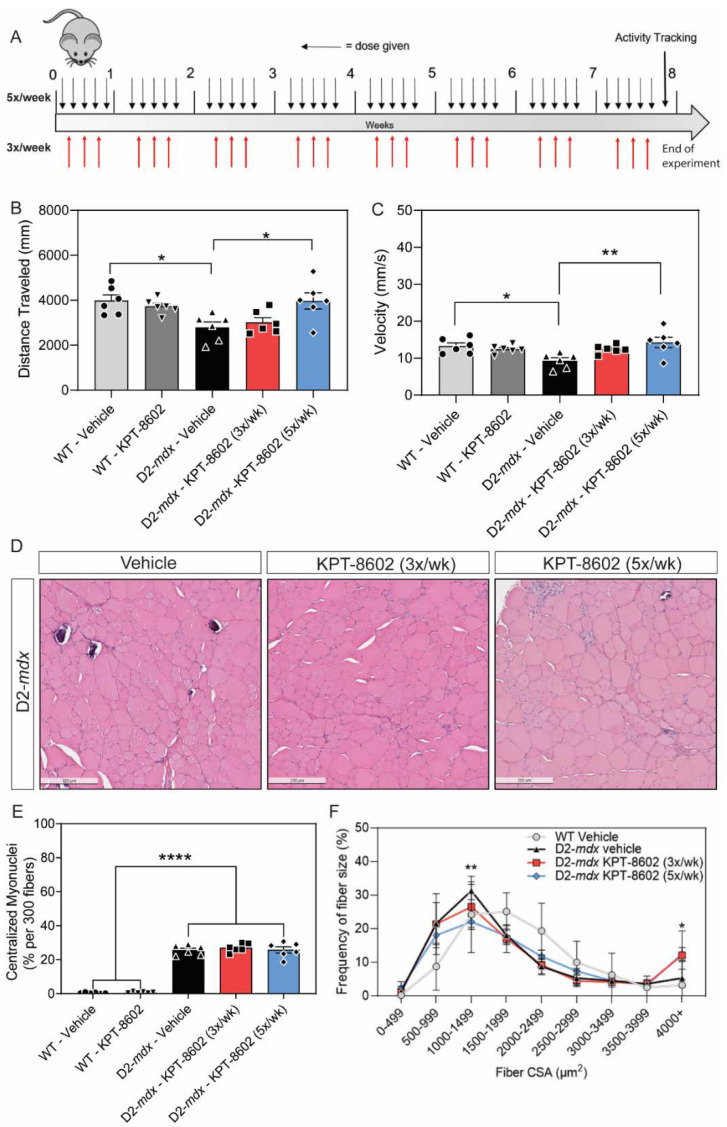
KPT−8602 treatment improves basal locomotion and histological markers in DMD mouse muscle. (**A**) Schematic of mouse experiments illustrating the timeline of dosage in each condition and activity tracking (**B**,**C**). Graphs summarizing total distance traveled (**B**) and average velocity (**C**) in an open field test. KPT−8602 treatment in D2-*mdx* mice significantly increased total distance travelled and average velocity compared to vehicle-treated D2-*mdx* mice (data is presented as mean ± SEM, *n* = 6; * *p* < 0.05, ** *p* < 0.01; one-way ANOVA with Tukey’s HSD test); (**D**) Representative images of tibialis anterior (TA) muscles stained with H&E across D2-*mdx* mice treated with either vehicle, 3x/week KPT−8602 or 5x/week KPT−8602. Scale bar = 200 µm; (**E**) KPT−8602 treatment did not reduce the number of centralized myonuclei, which is significantly increased in DMD pathology (data is presented as mean ± SEM, *n* = 6; **** *p* < 0.0001; one-way ANOVA with Tukey’s HSD test); (**F**) Fiber size frequency distribution curves of the cross-sectional area of myofibers revealed that KPT−8602 treatment decreased the number of small fibers and increased number of large fibers, indicative of a hypertrophy response compared to D2-*mdx* vehicle (data points are presented as mean ± SEM, *n* = 6; * *p* < 0.05, ** *p* < 0.01; one-way ANOVA with Tukey’s HSD test).

D2-*mdx* mice show decreased muscle mass and decreased force transmission in the skeletal muscle as early as 7 weeks [38,39]. To test the efficacy of KPT−8602 in dystrophic mice we performed oral administration of KPT−8602 in eight-week-old D2-*mdx* mice that were treated for 2 months at two separate dosing regimens. The first regimen was 3x/week at 5 mg/kg body weight) while the second was 5x/week at 5 mg/kg body weight with a single vehicle cohort serving as an internal control (Figure 2A). These dosing treatment regimens were selected based on previous studies with KPT−350 treatment, where 3x/week treatment of KPT−350 (5 mg/kg) resulted in significant reduction of dystrophic pathology in D2-*mdx* mice [27]. KPT−8602 was formulated to have a more-reversible binding affinity to XPO1 along with less ability to cross the blood–brain barrier, with the intent that it can be treated more frequently than KPT−350 [40]. Overall locomotor basal activity improved within the 5x/week treatment cohort of D2-*mdx* mice, which was comparable to WT levels of basal activity (Figure 2B,C). Specifically, 5x/week KPT−8602 treatment significantly increased total distance traveled (Figure 2B), and average velocity (Figure 2C) compared to vehicle-treated D2-*mdx* mice. Interestingly, while 3x/week KPT−8602 treatment slightly improved these parameters the 5x/week dosing regimen was overall better at ameliorating dystrophic locomotive deficits (Figure 2B,C). We performed a histological analysis on the tibialis anterior (TA) muscle harvested from all cohorts (Figure 2D). Consistent with previous studies, the vehicle-treated D2-*mdx* cohort had increased centralized myonuclei, a biomarker of regeneration, compared to WT TA muscle (Figure 2E) [41,42]. Interestingly, neither dose regimen of KPT−8602 affected the number of centralized myonuclei in D2-*mdx* mice (Figure 2E), however both dosing regimens significantly affected the fiber size frequency distribution curve (Figure 2F). In vehicle-treated D2-*mdx* mice, there is a large frequency of smaller fibers, indicative of the degenerative/regenerative cycles consistent with DMD muscle pathology [38]. KPT−8602 treatment, in particular the 5x/week dosing regimen, shifted the fiber size distribution, as evident by larger, more hypertrophic myofibers (Figure 2F). These findings were reflective of our previous findings demonstrating that KPT−350 also resulted in larger, more hypertrophic dystrophic myofibers [27]. Additionally, these findings may be a result of the blockade of NF-_Κ_B signaling by KPT−8602-mediated XPO1 inhibition, which has been shown to be a viable strategy for increasing myofiber size and reducing overall dystrophic pathologies [43,44].

### 3.3. KPT−8602 Improves Immunological Profiles in Dystrophic Mouse Muscles

**Figure 3:**
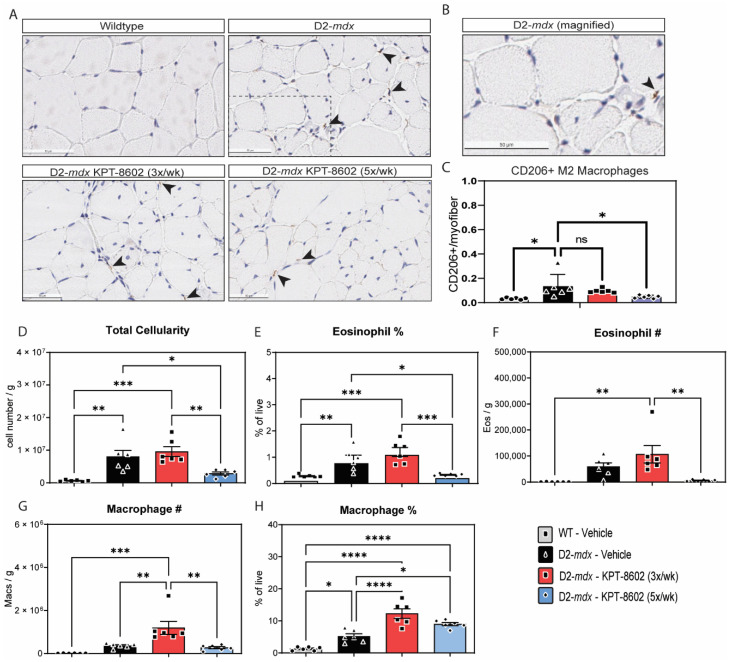
KPT−8602 treatment shifts D2-mdx muscles to a pro-regenerative, M2 macrophage profile. (**A**) Immunostaining of CD206-positive TA muscles from WT-vehicle, D2-*mdx* vehicle, D2-*mdx* KPT−8602 3x/week, and D2-*mdx* 5x/week experimental mouse cohorts. Arrows indicate CD206 stained macrophages. Boxed area magnified in B. Scale bar = 50 µm; (**B**) Magnified inset of a CD206+ macrophage from the D2-*mdx* sample; (**C**) Quantification of the total number of CD206+ M2 macrophages normalized to the total number of myofibers per area quantified to a minimum of 500 myofibers. All data is presented as mean ± SEM, *n* = 6; * *p* < 0.05, ** *p* < 0.01, *** *p* < 0.001, **** *p* < 0.0001; one-way ANOVA with Tukey’s HSD test); (**D**) Total cellularity measured in quadricep muscle cell preparations from the 4 experimental cohorts: WT vehicle (grey), D2-*mdx* vehicle (black), D2-*mdx* 3x/week (red); D2-*mdx* 5x/week (blue); (**E**,**F**) graphs showing the percentage of eosinophils and the total eosinophil number/gram muscle wet weight; (**G**,**H**) Graphs showing the percentage of macrophages and the total macrophage number/gram muscle wet weight.

We next examined the effect of KPT−8602 on the cellular composition of dystrophic muscle. KPT−8602 treatment 3x/week had no effect on the overall cellularity of muscle single-cell suspensions prepared from D2-*mdx* (Figure 3D). However, D2-*mdx* treated 5x/week with KPT−8602 have significantly reduced total cellularity compared to vehicle-treated mice (Figure 3D). We interrogated the myeloid cell composition by flow cytometry and found that the frequency (Figure 3E) and absolute number (Figure 3F) of eosinophils was significantly reduced in D2-*mdx* treated 5x/week with KPT−8602 compared to mice treated 3x/week. KPT−8602 treatment at 3x/week did not influence eosinophil frequency or numbers compared to vehicle-treated D2-*mdx* mice.

KPT−8602 increased the proportion of macrophages when D2-*mdx* mice were treated 3x/week (Figure 3F). Although KPT−8602 administered 5x/week only slightly reduced the frequency of macrophages compared to D2-mdx mice treated 3x/week, it significantly reduced the number of total macrophages (Figure 3H). In prior studies, we found that KPT−350 increased CD206+ M2 macrophages [27]. Therefore, we enumerated CD206+ macrophages by immunohistochemistry, to determine whether the modulation of macrophage frequency and number by KPT−8602 in D2-*mdx* mice were attributed to changes in the CD206+ macrophage subpopulation. Representative images of immunohistochemical staining of CD206 revealed that CD206+ macrophages are elevated in D2-*mdx* mice treated with KPT−8602 3x/week but not mice treated 5x/week (Figure 3C). Total macrophages were quantified using F4–80 staining. There was no significant change in the amount of CD206- macrophages (Supplemental Figure S3). The quantification of CD206+ macrophages suggests that modulation of total macrophages by KPT−8602 is partly attributed to a reduction in CD206+ macrophages (Figure 3A,B). Additionally KPT−8602 treatment had no effect on the amount of fibrosis in either the 3x/week or 5x/week dose (Supplemental Figure S4).

### 3.4. KPT−8602 Reduced Osteopontin Serum Levels in D2-Mdx Mice

**Figure 4:**
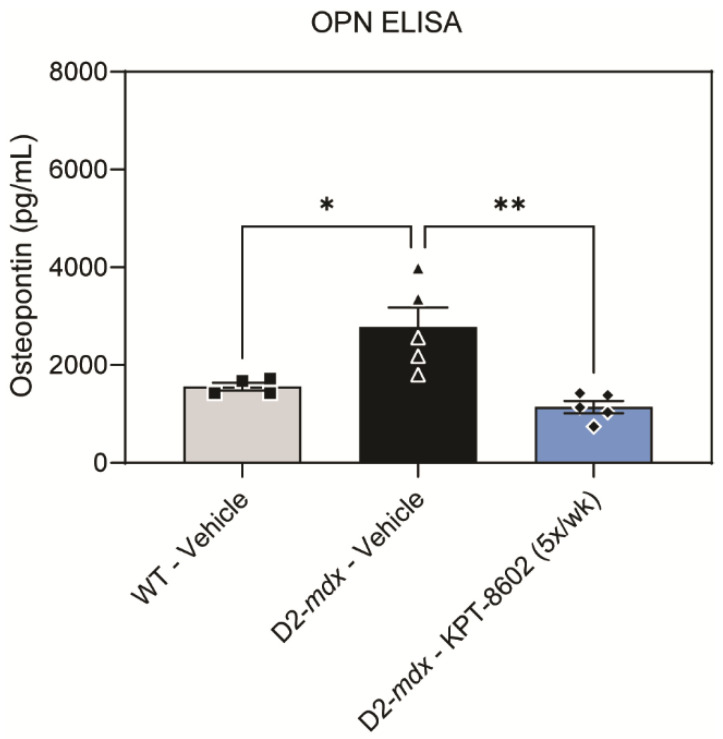
KPT−8602 reduces osteopontin serum levels in D2-*mdx* mice. Osteopontin (OPN) in circulating serum levels was assessed by ELISA assay. KPT−8602 (5x/week) treatment significantly reduced osteopontin levels compared to vehicle-treated D2-*mdx*, demonstrating levels comparable to WT mice (data is presented as mean ± SEM, *n* = 4–5 mice per cohort; * *p* < 0.05, ** *p* < 0.01; one-way ANOVA with Tukey’s HSD test).

OPN is a critical inflammatory DMD serum biomarker that is secreted by dystrophic inflammatory cells to drive fibrosis and inflammation, and extra-cellular matrix remodeling [45]. Thus, we investigated the effects of KPT−8602 on OPN protein levels in serum. As has been previously established, the vehicle-treated D2-*mdx* had significantly higher levels of serum osteopontin compared to vehicle-treated WT (Figure 4). The KPT−8602 5x/week dosing regimen reduced OPN to a level comparable to WT (Figure 4). These findings demonstrate that oral KPT−8602 administration in DMD mice effectively blocks a known serum biomarker of DMD muscle as a consequence of altering solely the DMD immunological profile.

## 4. Discussion

Our findings demonstrate that the SINE compound KPT−8602 is effective at reducing dystrophic symptoms and overall pathologies in DMD zebrafish and mouse models. The 5x/week KPT−8602 dosing regimen in the D2-*mdx* mice showed efficacy similar to that of previously reported findings of KPT−350 [27]. The use of XPO1 inhibition to block anti-inflammatory and anti-fibrotic signaling in dystrophin-deficient muscle remains a viable option as a combinatorial therapy that could be used with other dystrophin-replacement and exon-skipping compounds. Recently, using the combinatorial approach of oral administration of dantrolene and exon-skipping anti-sense oligonucleotides (AONs) in *mdx* mice has been shown to be effective in blocking DMD muscle pathologies [46]. Other compounds such as using an ActRIIB:ALK4-Fc neutralizing compound with a phosphorodiamidate morpholino oligomer (PMO) was also shown to block dystrophic pathologies in *mdx* mice [47]. One can envision combining KPT−8602 with an exon-skipping PMO or a µ-dystrophin adeno-associated viral gene therapy strategy to strengthen the myofiber membrane integrity while blocking inflammatory signaling and immune cell infiltration in DMD muscles.

Several studies have demonstrated a critical role for immune cell populations in dystrophin-deficient muscle and the amplification of dystrophic pathologies [48,49,50]. T-cells and macrophage profiling of DMD mouse muscles have shown that pro-regenerative and pro-inflammatory cells exist in dystrophic muscle and contribute to the progression of DMD [37]. Additionally, one of the immune cell-secreted cytokines that has been shown to promote fibrosis and inflammatory signaling in dystrophin-deficient muscles, osteopontin, was shown to be reduced in circulating serum levels upon KPT−8602 treatment [51,52,53]. *Osteopontin* (*SPP1*) genetic variants have been identified in DMD patient cohorts and shown to be a predictor of the age of loss-of-ambulation in DMD patients [54,55]. Indeed, both genetic ablation of *osteopontin* has been shown to reduce dystrophic pathologies in *mdx* muscles, and subsequently anti-osteopontin compounds are being pursued for DMD therapies due to their ability to shift macrophages to a pro-regenerative phenotype [45,56]. We also demonstrated a shift towards a pro-regenerative macrophages and reduced circulating osteopontin levels after KPT−8602 treatment, which suggests that XPO1 inhibition regulates this pathway. Previous nuclear and cytoplasmic mass-spectrometry proteomic profiling of XPO1 protein cargos in thymoma cell lines yielded clues into what proteins were directly affected by XPO1 blockade [57]. Additional transcriptomic and proteomic analysis of XPO1 protein cargos in skeletal muscle may yield further insight into the exact mechanism(s) by which XPO1-blockage ameliorates dystrophic muscles.

Together, our findings support the principle of blocking inflammation and fibrosis as a therapy for DMD. Given the reversibility of KPT−8602 binding, and manageable safety profile of this newer SINE formulation, KPT−8602 can be given orally at more frequent dosing to block inflammation and fibrosis independent of *DYSTROPHIN* genetic mutation and age of DMD disease onset. Other neuromuscular disorders that have muscle inflammation and fibrosis (e.g., sarcoglycanopathies and certain limb-girdle muscular dystrophies) could also potentially benefit from KPT−8602 administration. As newer exon-skipping and gene therapy strategies emerge for these neuromuscular disorders, KPT−8602 could be applied in a combinatorial approach to further improve dystrophic pathologies and prevent disease progression.

## Data Availability

All data reported in this manuscript are presented as source or aggregate data in this manuscript. Original specimens and non-aggregated data is available on request to the corresponding author.

## Supplementary Materials

The following supporting information can be downloaded at: https://www.mdpi.com/article/10.3390/biomedicines10102400/s1, Figure S1: Unaffected and affected sapje zebrafish larvae representative bright field and birefringence images taken at 6 dpf; Figure S2: Short-term KPT-8602 treatment showed no significant difference in sapje zebrafish larvae motility at 6 dpf; Figure S3: KPT-8602 treatment did not affect M1 macrophage number; Figure S4: KPT-8602 treatment did not significantly affect fibrotic area.
