# Supplementary material for: Next-Generation SINE Compound KPT−8602 Ameliorates Dystrophic Pathology in Zebrafish and Mouse Models of DMD
Source: Biomedicines. 2022 Sep 26;10(10):2400. doi: 10.3390/biomedicines10102400 (PMC9598711; doi:10.3390/biomedicines10102400)
Supplement: Supplementary file 1 [file biomedicines-10-02400-s001.zip › biomedicines-1899881-supplementary.pdf]

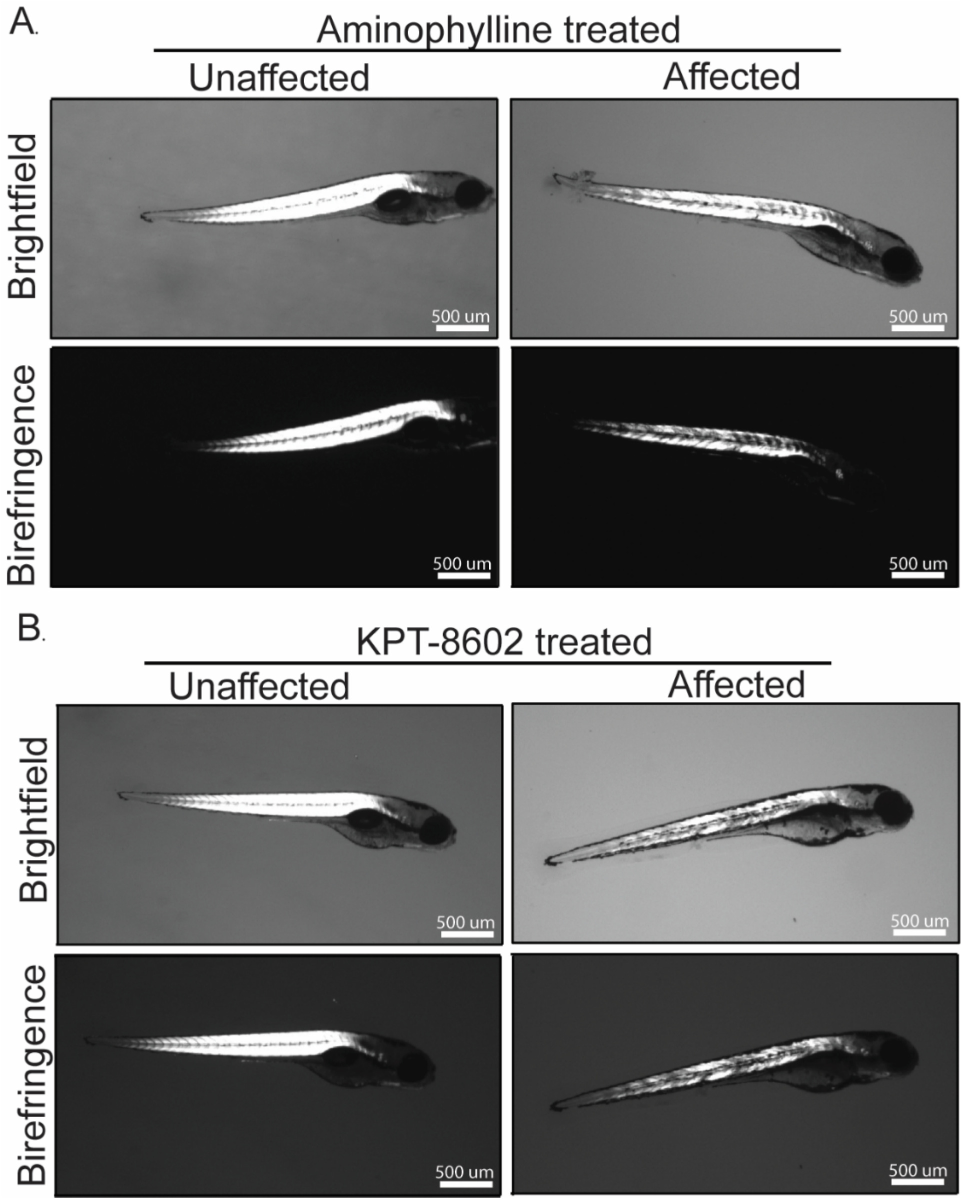

Supplemental Figure S1. Unaffected and affected *sapje* zebrafish larvae representative bright field and birefringence images taken at 6 dpf. A. and B. The *sapje* larvae were treated with either aminophylline or KPT-8602 at 2.5 µM starting at 1 dpf with drug water changed every other day. Scale bars represent 500 µm.

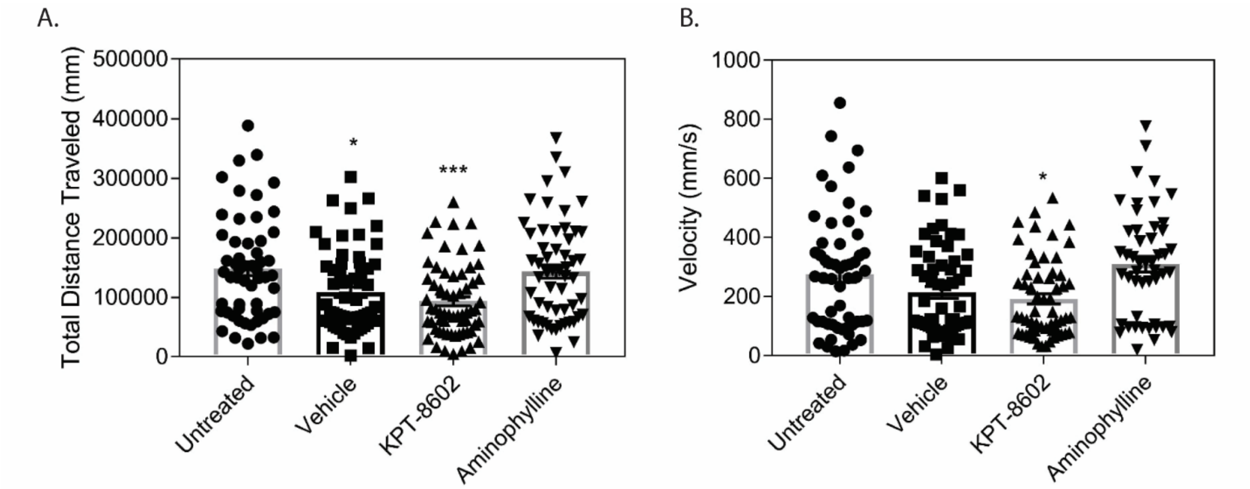

Supplemental Figure S2. Short-term KPT-8602 treatment showed no significant difference in *sapje* zebrafish larvae motility at 6 dpf. *sapje* zebrafish larvae were treated with either vehicle, KPT-8602 or aminophylline (2.5 μM) from 6 dpf. Untreated control cohort also shown. A. Total distance traveled in millimeters (mm). B. Average velocity (mm/s) were measured with the DanioVision motility tracker at 6 dpf (n=80 fish per cohort, error bars represent SEM). *p-value < 0.05 compared to untreated control cohort, *** p-value < 0.005. One-way ANOVA with Tukey's HSD (honest significant difference) test.

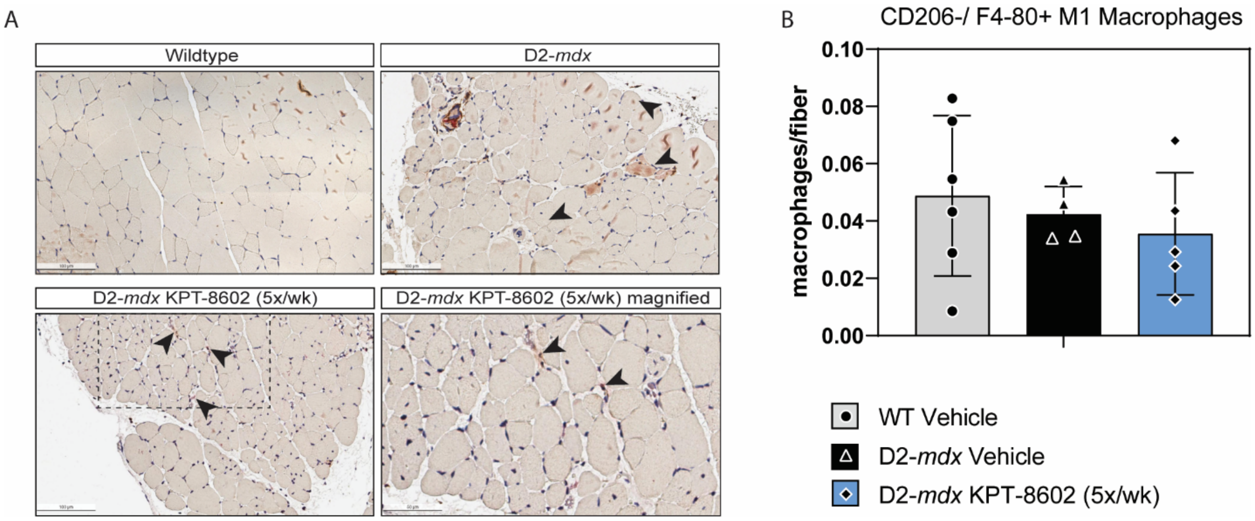

Supplemental Figure S3. KPT-8602 treatment did not alter M1 macrophage number. A. Representative images of tibialis anterior muscle immunohistochemically stained for F4-80. Black arrows indicated positively stained macrophages. Boxed inset magnified in lower right panel. Scale bars represent 100 μm or 50 μm in the magnified image. B. KPT-8602 treatment in D2-*mdx* mice did not significantly increase CD206- macrophages (data is presented as mean ± SEM, n =4-6; *p < 0.05, **p < 0.01; one-way ANOVA with Tukey's HSD test).

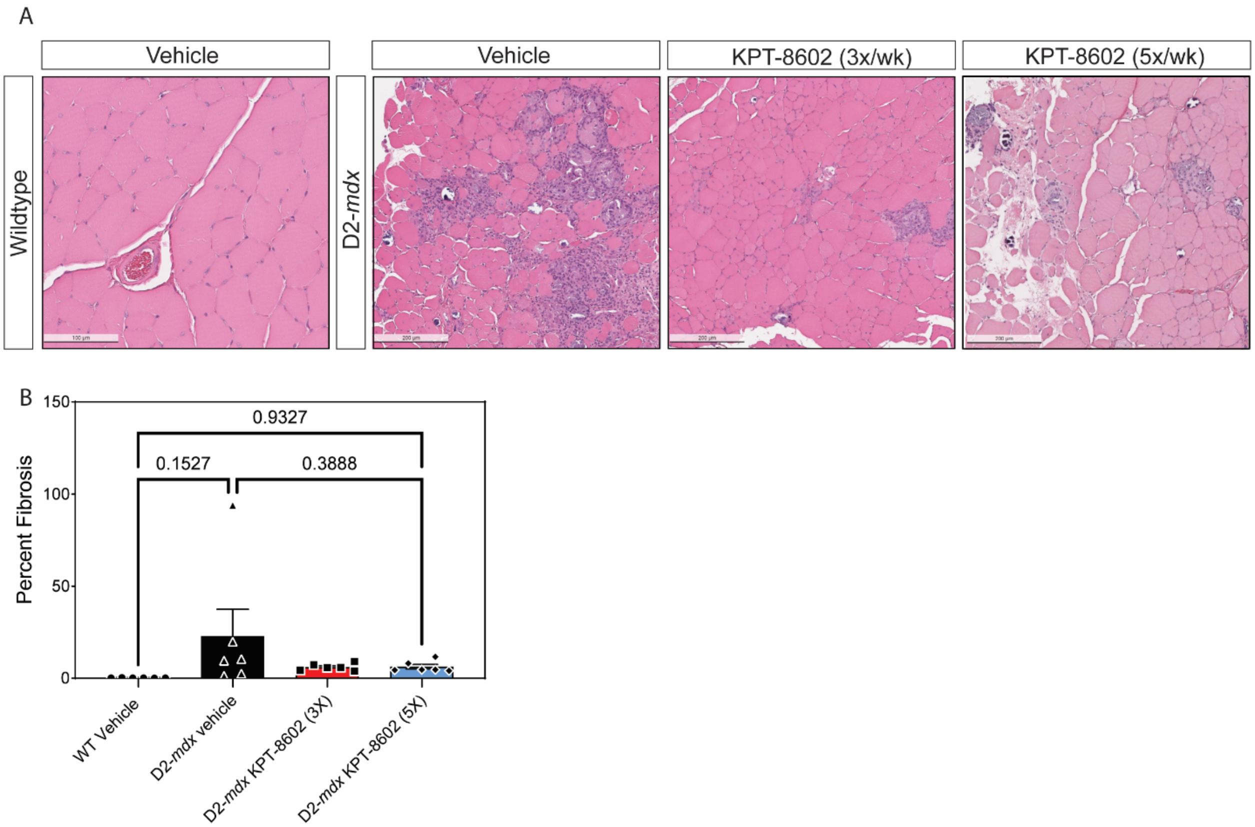

Supplemental Figure S4. KPT-8602 treatment did not significantly affect fibrotic area. A. Representative images of fibrotic area within tibialis anterior muscle stained with hematoxylin and eosin. Scale bar represents 200 µm. B. KPT-8602 treatment in D2-*mdx* mice did not significantly decrease fibrotic area (data is presented as mean ± SEM, n =6; *$p < 0.05$, **$p < 0.01$; one-way ANOVA with Tukey's HSD test).
